# Underlying structure in the dynamics of chase and escape interactions

**DOI:** 10.1038/s41598-019-51524-y

**Published:** 2019-10-21

**Authors:** Kazushi Tsutsui, Masahiro Shinya, Kazutoshi Kudo

**Affiliations:** 1 Graduate School of Arts and Sciences, The University of Tokyo, Toyko, Japan; 2 Graduate School of Integrated Arts and Sciences, Hiroshima University, Hiroshima, Japan; 3 Graduate School of Interdisciplinary Information Studies, The University of Tokyo, Toyko, Japan

**Keywords:** Decision, Perception, Human behaviour, Animal behaviour

## Abstract

Chase and escape behaviors are important skills in many sports. Previous studies have described the behaviors of the attacker (escaper) and defender (chaser) by focusing on their positional relationship and have presented several key parameters that affect the outcome (successful attack or defense). However, it remains unclear how each individual agent moves, and how the outcome is determined in this type of interaction. To address these questions, we constructed a chase and escape task in a virtual space that allowed us to manipulate agents’ kinematic parameters. We identified the basic strategies of each agent and their robustness to changes in their parameters. Moreover, we identified the determinants of the outcome and a geometrical explanation of their importance. Our results revealed the underlying structure of a simplified human chase and escape interaction and provided the insight that, although each agent apparently moves freely, their strategies in two-agent interactions are in fact rather constrained.

## Introduction

Chase and escape behaviors are fundamental skills in many sports and are crucial for the survival of many animals in the wild making them highly important behaviors^1–5^. A number of factors, such as strategy, kinematic ability, and surroundings, are involved in determining the outcome (i.e., successful escape or interception)^6,7^, which makes any explanation of these behaviors complex.

Geometrical models have provided a framework for determining the conditions for escape success^2,3,8–10^. For example, a model of the initial phase of escape behavior shows what escape angle is needed to reach a safety zone, based on the kinematic parameters such as speeds of prey and predator and distance between these two agents (timing of the escape response)^9^. In addition, models of aerial sequential escape behavior have shown that speeds, turn rates (minimum turning radii), and distance between agents are important determinants of success for prey attempting to reach a safety zone that flanks either side of an approaching predator^2,10^. Although these models make assumptions for simplicity, such as evader and pursuer move at constant speed, the predictions are consistent with observed escape behaviors in some cases^6,7,10^. These findings have shown the usefulness of geometric models, but the effectiveness of such models for human chase and escape behaviors are unknown.

**Figure 1:**
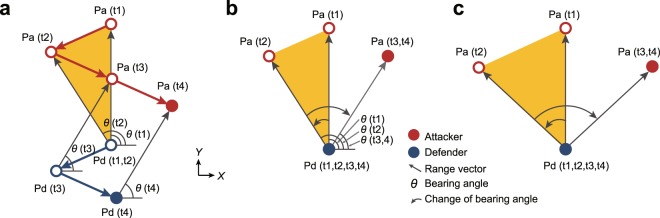
Geometrical models of attacker-defender interactions. (**a**) In the initial phase (t1 to t2; yellow section), the attacker moves from the starting position directly facing the defender. During this phase, the defender does not move as a result of visuomotor delay. In the second phase (t2 to t3), the attacker changes direction, but the defender moves in the opposite direction to the attacker because they are using perceptual information from the initial phase. In the third phase (t3 to t4), the defender changes direction to move in the same direction as the attacker. (**b**) When the attacker and defender move as shown in (**a**), in the initial phase, the bearing angle increases (in this Fig., to the left). In the second phase, the change in bearing angle is twice that of the change in the initial phase, because the attacker and defender move in opposite directions in the *X*-axis during this phase. As a result, during this phase, the change in the bearing angle increases to the right. In the third phase, the bearing angle does not change because the attacker and defender are moving in parallel. (**c**) In a case in which the speed of the agents is increased, in the initial phase, the attacker’s displacement during the visuomotor delay is greater, and thus the change in the bearing angle is greater than in (**b**). As in the case of (**b**), the change in the bearing angle in the opposite direction is twice as great during the second phase as during the initial phase, and the bearing angle does not change during the third phase.

In sports, chase and escape behaviors (i.e., one-on-one) have been studied, focusing on the positional relationship between two agents, attacker and defender^11–17^. In these studies, chase and escape behaviors have been described as the change of bearing angle, which is the angle between the range vector (defender to attacker) and the *X*-axis (mediolateral direction) in an absolute coordinate system (Fig. 1a). In sports, chase and escape behaviors are often performed by agents of similar speed and maneuverability, as players are generally matched with opponents belonging to the same category (e.g., junior-junior and professional-professional). Thus, once an attacker passes a defender, the attacker is rarely overtaken by the defender. As a result, we can assume that the escape success is achieved when the bearing angle becomes less than 0 degrees, or more than 180 degrees^18^. This approach can simplify complex chase and escape behaviors that involve multiple turns. However, although previous studies have presented certain kinematic parameters that could be important^16,19–22^, the relationship between these parameters and the determinants of chase and escape outcomes remains unclear.

**Figure 2:**
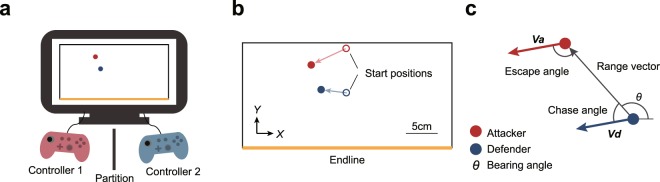
Experimental methods. (**a**) Participants were each seated in a chair and operated a joystick on the left side of a controller that controlled the velocity of a disk (red or blue) on a screen. A partition prevented direct viewing by each participant of the other’s hands. (**b**) The red disk, representing the attacker, started in the upper middle of the screen. The blue disk, representing the defender, started in the center of the screen. The diameter of each disk was 1.0 cm. The attacker’s goal was to reach the end line (a yellow line) located behind the defender without being caught (defined by contact between the outer edges of the disks) by the defender, whereas the defender’s goal was to catch the attacker. A black rectangle surrounding the disks defined the “court” area for the task. If the attacker left the bounds of the court, the trial was deemed a defensive success. (**c**) We defined a range vector, pointing from the defender to the attacker. The chase angle was defined as the angle between the range vector and the velocity vector of the defender. The escape angle was defined as the angle between the inverse of the range vector (i.e., a vector pointing from the attacker to the defender) and the velocity vector of the attacker. The bearing angle (*θ*) was defined as the angle between the range vector and the *X* axis. Finally, the inter-agent distance was defined as the magnitude of the range vector.

In this study, we examined the determinants of chase and escape outcomes, based on kinematic parameters. The key parameters, such as velocity (movement speed and direction), response time (visuomotor delay), and inter-agent distance, presented by the previous studies are geometrically associated with changes in the bearing angle. In the initial phase of a maneuver, the displacement of the attacker during the defender’s response time is determined by the product of the velocity of the attacker and the response time of the defender (illustrated by the yellow section in Fig. 1a). It is obvious that this displacement changes the bearing angle (Fig. 1b). In the later phase (where the two agents move), the change in bearing angle depends on the displacement of both the attacker and the defender. The change is smaller when agents move in the same mediolateral direction, and larger when agents move in the opposite mediolateral directions (see the second and third phases in Fig. 1a,b). An opposite movement in the mediolateral direction is caused by the defender’s visuomotor delay. The visuomotor delay, which is the latency from sensory input to motor output, is inevitable in animals and is approximately constant in humans^23^. Thus, if the speeds are larger, the displacements during the response time and the changes in the bearing angle should be larger (Fig. 1c). That is, increased speed should make the bearing angle closer to the condition of escape success in which the bearing angle is less than 0 degrees or more than 180 degrees. In other words, the increase in speed can be advantageous for the attacker. Similarly, the increase in the response time should make the bearing angle closer to the condition of escape success and can also be advantageous for the attacker. To test these possibilities, we constructed a virtual chase-and-escape task (Fig. 2a), which allows us to manipulate the kinematic parameters of the agents. Thus, it is possible to examine the effects of certain manipulated parameters on individual behaviors and interaction outcomes. Specifically, we manipulated the speeds of agents in Experiment 1, and the response time of defenders in Experiment 2. In both experiments, the attacker was required to move past the defender and reach the end line behind the defender, and the defender was required to catch the attacker (Fig. 2b). In the first experiment, we showed that the escape (attack) success is more frequent with increase in speed. Then, in the second experiment, we confirmed that the escape success is more frequent with increase in response time.

The contributions of this study are (i) to show the importance of the relationship between speed and visuomotor delay in chase and escape interactions in sports, (ii) to obtain suggestions that these parameters may determine the outcome of situations in which evaders make sharp turns, such as terrestrial chase and escape interactions in animals, and (iii) to provide the insight that seemingly unconstrained individual behavior may actually be quite constrained in two-agent interactions.

## Materials and Methods

### Ethics statement

The study was conducted in accordance with the Declaration of Helsinki and approved by the Ethics Committee of the University of Tokyo of Arts and Sciences. Informed consent was received from each participant before the experiments.

### Participants

Twelve healthy right-handed male students who exercised regularly participated in each experiment (mean age ± SD: Experiment 1, 24.9 ± 2.3; Experiment 2, 25.0 ± 2.4). Each participant received 1000 yen per hour as a reward. In both experiments, participants were recruited in pairs and each member of each pair took on the roles of both attacker and defender in turn.

### Experimental setup

Stimuli were presented in a dimly lit room on a 15.5 in (34.3 × 19.3 cm) screen. The participants were seated at a viewing distance of 50 cm. A partition prevented direct viewing of the other player’s hands. Data on the positions of the attacker and defender were recorded on a computer (Sony SVF152C16N) running Psychtoolbox 3.0 software at a frequency of 60 frames per second, and a resolution of 1366 × 768 pixels.

### Experimental design

Participants interacted with the task using Xbox One controllers (Fig. 2a,b). The dimensions of the court onscreen were 33.1 cm × 16.6 cm (width × height). Each participant controlled either a red disk representing an attacker or a blue disk representing a defender on the screen. The diameter of each disk was 1.0 cm. The objective of the attacker was to get past the defender and reach the end line (a yellow line) behind the defender, whereas the objective of the defender was to catch the attacker. We regarded a “catch” as a situation in which the outer edges of the disks were in contact. If the attacker left the bounds of the court, the trial was deemed a defensive success. The velocity of each agent was determined by the degree of inclination of the joystick on their respective controller. In preliminary experiments, we tested the agents at various speeds (e.g., attacker speed: defender speed = 1,0: 0.7, 0.8, 0.9, 1.0, 1.1, 1.2 or 1.3). If there were a speed difference between the agents, the faster one had a distinct advantage, and it was difficult for slower one to keep his or her motivation. Thus, in each experiment, we made the maximum speed of the attacker and the defender equal. The experimental task began with a start cue. No additional instruction, such as a time limit, was given to participants. To provide feedback on the result of each trial, when the attacker reached the end line (a successful attack), a high-pitched beep was played. Conversely, when the defender caught the attacker or the attacker left the bounds of the court (a successful defense), a low-pitched beep sounded. The number of successful attacks was indicated at the end of each block; blocks consisted of 30 trials. There were 40 warm-up trials and 240 experimental trials per pair of participants. For the experimental trials, each participant controlled the attacker and defender for 120 trials each. Experimental trials were presented in eight blocks. The role of each participant was randomized between blocks, and the experimental condition was randomized between pairs.

### Experimental conditions

There were two experimental conditions in each experiment. In Experiment 1, we manipulated the maximum speed of the agents (slow: 3 cm/s, vs. fast: 4.5 cm/s). In Experiment 2, we manipulated the delay between the defender’s joystick operation and movement of the onscreen agent (no-delay vs. added-delay). The duration of the delay in the added-delay condition was 133 ms. Both agents’ maximum speeds were set at 3 cm/s in both conditions in Experiment 2.

### Data analysis

All data analysis was performed in MATLAB (MathWorks). We analyzed only the data collected while the absolute angle between the defender and the attacker was in the range 0 to 180 degrees, to exclude situations in which the defender had given up trying to catch the attacker. The range vector was defined as the vector from the position of defender to that of attacker (Fig. 2c). The bearing angle was defined as the angle between the range vector and the *X*-axis in an absolute coordinate system. The chase angle was defined as the angle between the range vector and the velocity vector of the defender, and the escape angle was defined as the angle between the inverse of the range vector and the velocity vector of the attacker. The magnitude of the range vector corresponds to the inter-agent distance. The mean escape angle and mean chase angle were calculated as an average of the direction in which the attacker and defender moved in each instance, respectively, after pooling data from all trials. Each instance was defined by a pair of integers (the bearing angle and inter-agent distance in mean escape angle; and the bearing angle and escape angle in mean chase angle), and the parameters for each experimental data point were rounded to the nearest integer to bin the response into an instance. Response time was defined as the time between a directional change in the *X*-axis velocity of the attacker and a corresponding change in that of the defender. A directional change was identified by a change in the sign of the *X-*axis velocity, that is, from positive to negative, or vice versa. We limited the range of response times to 0 to 750 ms and excluded any response time exceeding 750 ms from analysis. A turning phase was defined as the 500 ms interval following a change in direction in the *X*-axis velocity of the attacker. A straight phase was defined as any other time during the trial.

### Statistical analysis

To test the relationship of the mean escape angles and mean chase angles between conditions (slow and fast), we calculated correlation coefficients. In the correlation analysis, we extracted data from all trials for situations in which the mean angle appeared in both conditions, and we used circular correlation coefficient *ρ* instead of Pearson’s product-moment correlation coefficient *r* because both values were circular^24^. For comparisons of the escape angle and chase angle between the conditions, we did not distinguish between the left and right in terms of the agent’s direction of movement relative to their opponent, instead using the absolute value of the angle. For comparisons of histograms of the *Y*-axis velocity of the defender between conditions, we used the Kolmogorov-Smirnov test. For comparison of the variables between conditions and phases (straight and turning), we used paired *t*-test if the normality assumption was accepted by Lilliefors test. If rejected, Wilcoxon signed-rank test was used. To test the relationship between the bearing angle and the velocity of the defender in the *Y*-axis, we did not distinguish between left and right in measuring the size of the bearing angle. That is, we calculated the Pearson’s product-moment correlation coefficient, *r*, between the degree of divergence from 90 degrees in the bearing angle and the displacement of the defender in the *Y*-axis. In this analysis, the possible values of the bearing angle were divided into nine bins of ten degrees each, and the average value of the defender’s *Y*-axis velocity in each bin was calculated for each participant. Effect sizes were estimated using Cohen’s *d* for *t*-test, and matched-pairs rank-biserial correlation *r* for Wilcoxon signed-rank test^25^; we report the absolute values of effect sizes. The statistical significance level was set at *p* < 0.05. Statistical analyses were performed using the MATLAB Statistical Toolbox (MathWorks).

## Results

### Experiment 1

In Experiment 1, we manipulated the agents’ speeds of movement using two conditions. The agents’ maximum speeds were set at 3 cm/s across the display in the slow condition, and 4.5 cm/s in the fast condition.

**Figure 3:**
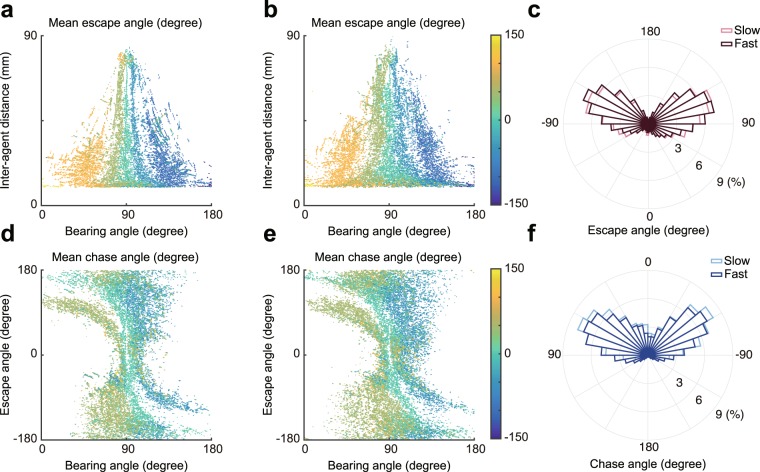
Directions of motion for attackers and defenders in each condition in Experiment 1. (**a**,**b**) Color represents the average escape angle for a given bearing angle and inter-agent distance, (**a**) in the slow condition and (b) in the fast condition. This is an average of the direction in which the attacker moved in each instance across all trials. (**c**) Escape angles were mostly obtuse in both conditions. (**d,e**) Color represents the average chase angle for a given bearing angle and escape angle, (**d**) in the slow condition and (e) in the fast condition. This is an average of the direction in which the defender moved in each instance across all trials. (**f**) Chase angles were mostly acute in both conditions.

Figure 3a,b shows the mean escape angle in each instance (see Materials and Methods for details) in the slow (3a) and fast (3b) conditions. The mean escape angle in each instance was highly correlated between the conditions (*ρ* = 0.88, *p* < 0.001). The majority of escape angles were obtuse (slow: 66%, fast: 68%), especially if the first three seconds in each trial were excluded (slow: 85%, fast: 83%), and there was no statistically significant difference in escape angle between the conditions (*t*_11_ = 0.73, *p* > 0.05, *d* = 0.30; Fig. 3c). The mean chase angle in each instance was correlated between the conditions (*ρ* = 0.60, *p* < 0.001; Fig. 3d,e). The majority of chase angles were acute (slow: 90%, fast: 83%), and there was no statistically significant difference in chase angle between the conditions (*t*_11_ = 1.95, *p* > 0.05, *d* = 0.79; Fig. 3f). These results indicated that the attacker and the defender largely moved in parallel in both conditions.

**Figure 4:**
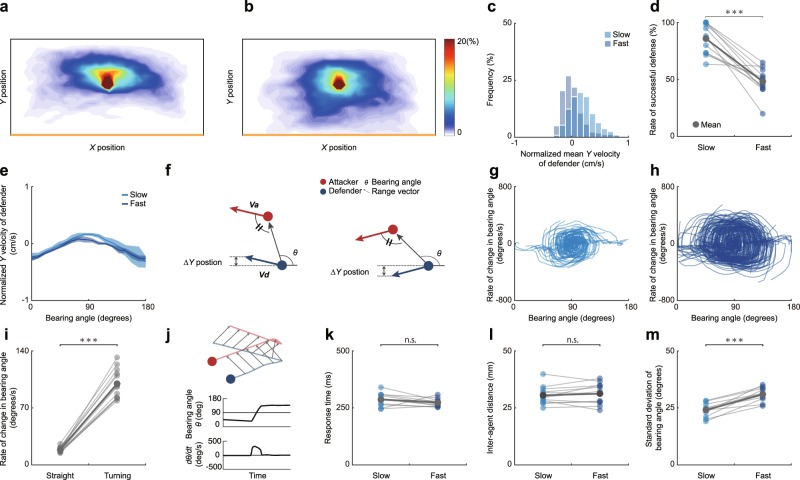
Comparison of behaviors between speed conditions in Experiment 1. (**a**,**b**) Heat maps showing the proportion of time spent in each location by the defender, (**a**) in the slow condition and (**b**) in the fast condition. (**c**) A histogram showing the distribution of the normalized mean *Y*-axis velocity of the defender, computed for each trial. The defender retreated toward the end line to a greater extent in the fast condition than in the slow condition. (**d**) The rate of successful defense was lower in the fast condition than in the slow condition. (**e**) The relationship between the bearing angle and the normalized *Y-*axis velocity of the defender. (**f**) Illustrations of the relationship between the normalized *Y*-axis velocity of the defender and the size of the bearing angle. The displacement of the defender in the *Y-*axis varies with the bearing angle, even though the escape angle and the chase angle remain the same. (**g**,**h**) Typical examples of phase portraits, (**g**) in the slow condition and (**h**) in the fast condition. Each Fig. consists of overlaid trajectories from a single experimental block (30 trials). (**i**) The rate of change in the bearing angle was greater during turning phases than during straight phases. (**j**) An illustration of part of the trajectories in an example trial, accompanied by time-series data on the bearing angle and its derivative in a trial. The variation in these parameters was large during turning phases. (**k**) The response time of the defender to the attacker did not differ significantly between conditions. (**l**) The inter-agent distance also did not differ significantly. (**m**) The SD of the bearing angle was greater in the fast condition than the slow condition.

Figure 4a,b are heat maps of the proportion of time the defender spent in each location, for each speed condition. The defender moved backward (toward the end line) to a greater extent in the fast condition than the slow condition (*D* = 0.36, *p* < 0.001; Fig. 4c), and the successful interception rate was lower in the fast condition than the slow condition (*t*_11_ = 7.37, *p* < 0.001, *d* = 3.02; Fig. 4d). Subsequently, we explored why these differences occurred between the conditions. We found that whether the defender advanced or retreated was associated with the bearing angle (slow: *r* = 0.78, *p* < 0.001; fast: *r* = 0.90, *p* < 0.001; Fig. 4e,f). The phase portraits of the bearing angle and its derivative in a representative example are presented in Fig. 4g,h, and indicate less attraction to the center of the plot in the fast condition than in the slow condition. The rate of change in the bearing angle was greater during turning phases (i.e., when the agent was changing direction) than during straight movement phases (*W* = 78, *p* < 0.001, *r* = 1.0; Fig. 4I,j). There was no statistically significant difference between conditions in the response time of the defender (*t*_11_ = 1.43, *p* > 0.05, *d* = 0.59; Fig. 4k) or the inter-agent distance (*t*_11_ = 0.87, *p* > 0.05, *d* = 0.16; Fig. 4l). Consequently, the variance in the bearing angle was greater in the fast condition than the slow condition (*t*_11_ = 8.17, *p* < 0.001, *d* = 2.41; Fig. 4m).

### Experiment 2

**Figure 5:**
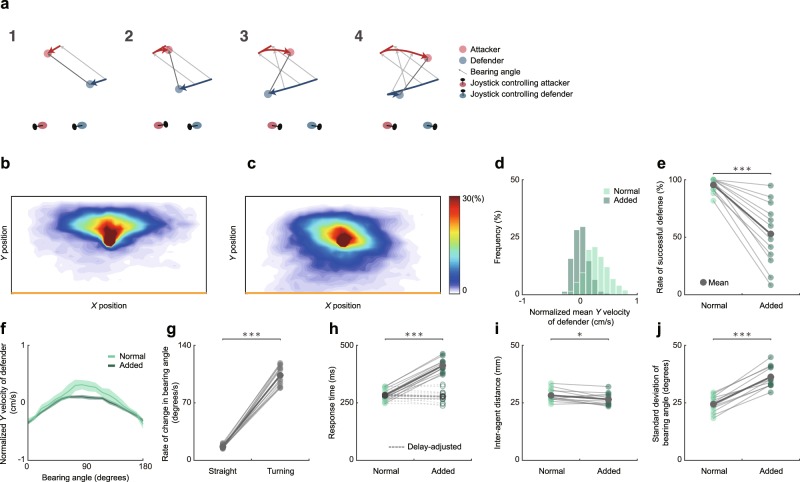
Comparison of behaviors between delay conditions in Experiment 2. (**a**) Illustration of the added-delay manipulation. (1) The attacker and the defender each move toward the left side. (2) The attacker changes direction from left to right in conjunction with the participant’s operation of the joystick. (3) The participant controlling the defender responds (by operating the joystick) to the attacker’s change in direction following the visuomotor delay. However, their operation of the joystick is not reflected immediately in the defender’s movement. (4) The defender now changes direction from left to right. The elapsed time between the attacker’s change in direction and the defender’s change in direction is the sum of the visuomotor delay and the additional, artificially-added delay. (**b**,**c**) Heat maps showing the proportion of time spent in each location by the defender, (b) in the normal-delay condition and (**c**) in the added-delay condition. (**d**) A histogram showing the distribution of the normalized mean *Y*-axis velocity of the defender, computed for each trial. (**e**) The rate of successful defense was lower in the added-delay condition than in the normal-delay condition. (**f**) The relationship between the bearing angle and the normalized *Y*-axis velocity of the defender. (**g**) The rate of change in the bearing angle was greater during turning phases than during straight phases. (**h**) Response time was increased when an artificial delay was added (see Methods). However, there was no significant difference between the conditions when the duration of the artificially-added delay was subtracted. (**i**) The inter-agent distance did not differ significantly between conditions. (**j**) The SD of the bearing angle was greater in the added-delay condition than the normal-delay condition.

We conducted a second experiment consisting of a normal-delay condition and an added-delay condition. In both conditions, the maximum speed of both agents was set at 3 cm/s. In the added-delay condition, we artificially added a 133 ms delay to the defender’s response. Specifically, the delay occurred between the participant’s operation of the joystick and the movement of the defender on the screen (Fig. 5a).

Figure 5b,c show heat maps of the defender’s position. The defender moved backward to a greater extent in the added-delay condition than the normal-delay condition (*D* = 0.59, *p* < 0.001; Fig. 5d), and the successful interception rate was also lower in the added-delay condition (*W* = 78, *p* < 0.001, *r* = 1.0; Fig. 5e). The *Y*-axis displacement of the defender was associated with the bearing angle for both conditions (normal-delay: *r* = 0.66, *p* < 0.001; added-delay: *r* = 0.89, *p* < 0.001; Fig. 5f). The rate of change in the bearing angle was greater during turning phases than during straight movement phases (*W* = 78, *p* < 0.001, *r* = 1.0; Fig. 5g). Although the defender’s response time was increased in the added-delay condition (*t*_11_ = 34.40, *p* < 0.001, *d* = 4.79), there was no statistically significant difference between the conditions once this artificial addition was subtracted (*t*_11_ = 1.30, *p* > 0.05, *d* = 0.18; Fig. 5h). The inter-agent distance was significantly smaller in the added-delay condition than in the normal-delay condition (*t*_11_ = 2.78, *p* < 0.05, *d* = 0.64; Fig. 5i), and the variance in the bearing angle was greater in the added-delay condition than in the normal-delay condition (*t*_11_ = 13.14, *p* < 0.001, *d* = 2.90; Fig. 5j).

## Discussion

We investigated how escaper (attacker) and chaser (defender) move and what determines the outcome of a human chase and escape behavior. We found that the attacker and defender moved in parallel during straight phases of movement, whereas their positional relationship (the bearing angle) changed during turning phases. This change in the bearing angle was crucial for each agent’s success, and an increase in each of the two parameters manipulated here, namely the speed of movement of the agents (Experiment 1) and the defender’s response time (Experiment 2), induced a larger change in the bearing angle.

In this study, the attacker’s goals were (i) to avoid being caught by the defender and (ii) to reach the end line behind the defender. The attackers largely moved at an obtuse angle with respect to the position of the defender. This strategy was in line with the first goal, but seems to work against the second goal. It should be noted that the attacker was able to simultaneously satisfy both the objectives by tilting the bearing angle on trials in which interception was successfully evaded (see right panel of Fig. 4f).

Conversely, the defender’s goals were (i) to prevent the attacker from breaking past them to reach the end line and (ii) to catch the attacker. One way to achieve these two objectives was to adopt the strategy of maintaining a constant bearing angle. In this strategy, the defender takes a trajectory such that the bearing angle remains constant, while their distance from the attacker is reduced^26,27^. This strategy should theoretically be impossible to beat if the defender can move at a speed equal to or faster than the attacker, without a visuomotor delay. Indeed, many animal species actually use this strategy in hunting prey^28–31^. Our finding that the defender tended to move in parallel with the attacker suggests that our human defenders also used this strategy, but they could not reduce the inter-agent distance because the maximum speeds of the agents were equal in this task.

The individual behaviors adopted by the agents were independent of the agents’ speeds and the defender’s response time. Previous research has shown that the relative velocity and inter-agent distance affect the outcome of chase and escape interactions in sports^16,19–21^. In Experiment 1, we manipulated the maximum speeds of the agents, and observed that an increase in speed increased the relative velocity during turning phases. In these cases, there are two geometrically possible ways for the defender to reduce (or prevent) this change in the bearing angle. One is to increase the inter-agent distance, and the other is to reduce their response time (e.g., by predicting the attacker’s changes of direction and responding accordingly). However, our results showed that changes in the inter-agent distance and response time were inadequate to offset the effect of a change in the agents’ speeds. These results suggest that, in this type of interaction, agents’ behaviors are constrained by agent-environment interactions^32–34^, and few possible strategies may be available. Despite this, in competitive situations, humans seem to decide on their strategy for themselves from numerous choices that they could make.

Our manipulation of agents’ speeds directly affected the outcome of chase and escape interactions, even though individual agents’ behaviors were unchanged. Relative velocity, a key parameter, is the difference between the velocities of the agents. It is obvious that the faster agent is at an advantage when there is a difference in speed, but even when both speeds were the same, larger absolute speed values gave a greater the advantage to the attacker. This advantage arises from the relationship between speed of movement and visuomotor delay. During turning phases, the defender responds to the attacker’s change in direction, and there is a delay between perception of the attacker’s change in direction and initiation of a movement in response^35–39^. This delay between a sensory input and a motor output arises as a result of neural conduction and transmission in the sensorimotor system, and a latency of 200–300 ms is inevitable in humans^23^. During the period of the visuo-motor delay, the defender moves in the opposite direction to the attacker, and as agents’ maximum absolute speed is increased, they can move further in the opposite direction over this period. Consequently, when the inter-agent distance is held constant, the change in the bearing angle during turning phases increases with the absolute speed of movement of the agents. As a result, attackers could be more likely to escape at higher speeds.

A similar change in the chase and escape interaction was observed with the manipulation of the defender’s visuomotor delay. As mentioned above, during turning phases the agents moved in opposite directions. Here, the displacement between the agents is equal to their velocities multiplied by the defender’s response time. In other words, both an increase in the agents’ speeds of movement and an increase in the defender’s response time cause an increase in the displacement. An increase in the defender’s response time is geometrically comparable to an increase in the agents’ speeds of movement during this phase of displacement, and could cause a similar change to chase and escape outcomes. In Experiment 2, we manipulated the defender’s response time. As expected, the outcomes of their behaviors were affected in a similar way to Experiment 1 (compare Figs 4a,b, 5b,c). These results suggest that agents’ speeds of movement and defenders’ visuomotor delay (response time) are comparable in a chase and escape interaction.

Our approach makes it possible to observe chase and escape behaviors in controlled situations of kinematic parameters, while these situations differ in some respects from the more complex chase and escape behaviors, such as those performed in a real environment. Firstly, in this study, an attacker and a defender were represented as disks onscreen. As many studies on sports have revealed, kinematic information relating to body parts is important, particularly in turning phases. For example, attackers try to deceive defenders with yawing upper body movements^39^, whereas defenders try to predict the directional change of the attacker using the attacker’s center of mass^38,40^. These differences may affect the variation in the response time including prediction. Secondly, in our approach, agents can always move at the same speed in any direction. As many researchers have shown, real chase and escape behaviors may be influenced by mechanical constraints such as morphological characteristics^41,42^, the preparatory state of the body^20,35^, and the relationship between mass and speed^43^. Finally, energy consumption for the attacker and defender in our virtual task is small, which are unlike real chase and escape behaviors^44–47^. For more detailed understanding, incorporating these factors would be necessary.

In conclusion, we found a simple structure in the dynamics of a chase and escape interaction, even though such dynamics appear to be complex. This simplicity arises from the consistency of individual behaviors when constrained by multiple requirements. As a result, the outcome of agents’ behaviors is substantially dependent on their parameters of maneuverability. These findings may provide a comprehensive link between individual behaviors and the outcome of their interactions.

## Data Availability

The datasets generated and analyzed in this study are available from the corresponding authors on reasonable request.
